# Supplementary material for: High-performance 2D electronic devices enabled by strong and tough two-dimensional polymer with ultra-low dielectric constant
Source: Nat Commun. 2024 Dec 30;15:10780. doi: 10.1038/s41467-024-53935-6 (PMC11685926; doi:10.1038/s41467-024-53935-6)
Supplement: Supplementary file 1 — Supplementary Information [file 41467_2024_53935_MOESM1_ESM.pdf]

Supplementary Information

High performance 2D electronic devices enabled by strong and tough two-dimensional polymer with ultra-low dielectric constant

Qiyi Fang[1,2,#] Kongyang Yi[3,#], Tianshu Zhai [1,#], Shisong Luo[4], Chen-yang Lin[1], Qing Ai[1], Yifan Zhu[1], Boyu Zhang[1], Gustavo A. Alvarez[5], Yanjie Shao[6], Haolei Zhou[2], Guanhui Gao[1], Yifeng Liu[1], Rui Xu[1], Xiang Zhang[1], Yuzhe Wang[2], Xiaoyin Tian[1], Honghu Zhang[7], Yimo Han[1], Hanyu Zhu[1], Yuji Zhao[4], Zhiting Tian[5], Yu Zhong[2], Zheng Liu[3,*], Jun Lou[1,*]

[1]Department of Materials Science and NanoEngineering, Rice University, Houston, TX 77005,USA.

[2]Department of Materials Science and Engineering, Cornell University, Ithaca, NY 14853, USA.

[3]School of Materials Science and Engineering, Nanyang Technological University, Singapore 639798, Singapore.

[4]Department of Electrical and Computer Engineering, Rice University, Houston, TX 77005, USA.

[5]Sibley School of Mechanical and Aerospace Engineering, Cornell University, Ithaca, NY 14853, USA.

[6]Department of Electrical Engineering and Computer Science, Massachusetts Institute of Technology, Cambridge, MA 02139, USA

[7]National Synchrotron Light Source II, Brookhaven National Laboratory, Upton, NY 11973, USA.

[#]These authors contribute equally: Qiyi Fang, Kongyang Yi, Tianshu Zhai

*Correspondence: jlou@rice.edu, z.liu@ntu.edu.sg

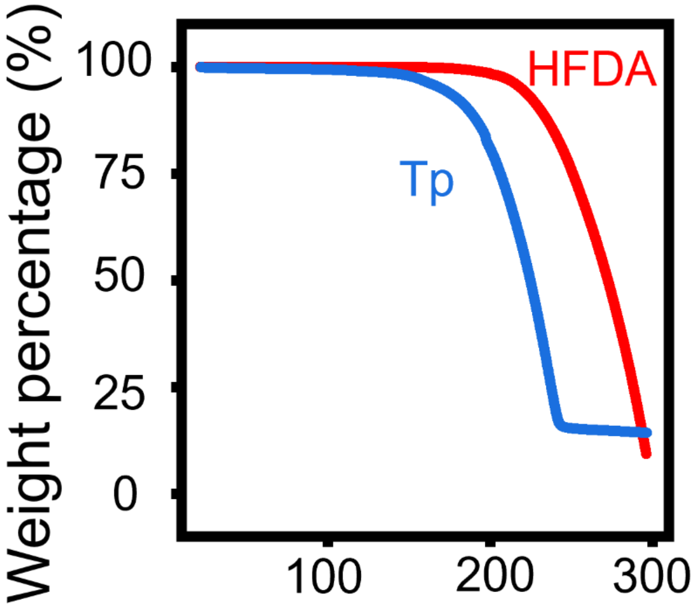

Supplementary Figure 1. TGA curves of Tp and HFDA. Both monomers begin to lose weight below 300℃.

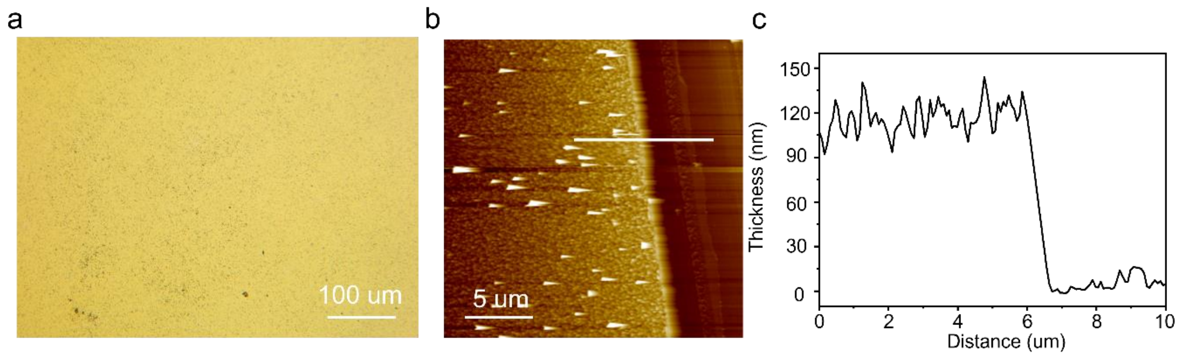

Supplementary Figure 2. (a) Optical image of solution grown 2DP-F. In brief, 2ml solutions of Tp (2mM) and HFDA(3mM) in acetonitrile were mixed and 0.2 ml of 6M AcOH was added. A desired substrate (sapphire in this case) was added into the solution and kept at room temperature for 3 days. A yellow film of 2DP-F on sapphire was picked out, washed with Acetonitrile for 3 times and dried under ambient for further characterization. (b) AFM image of solution grown 2DP-F film and (c) corresponding line profile.

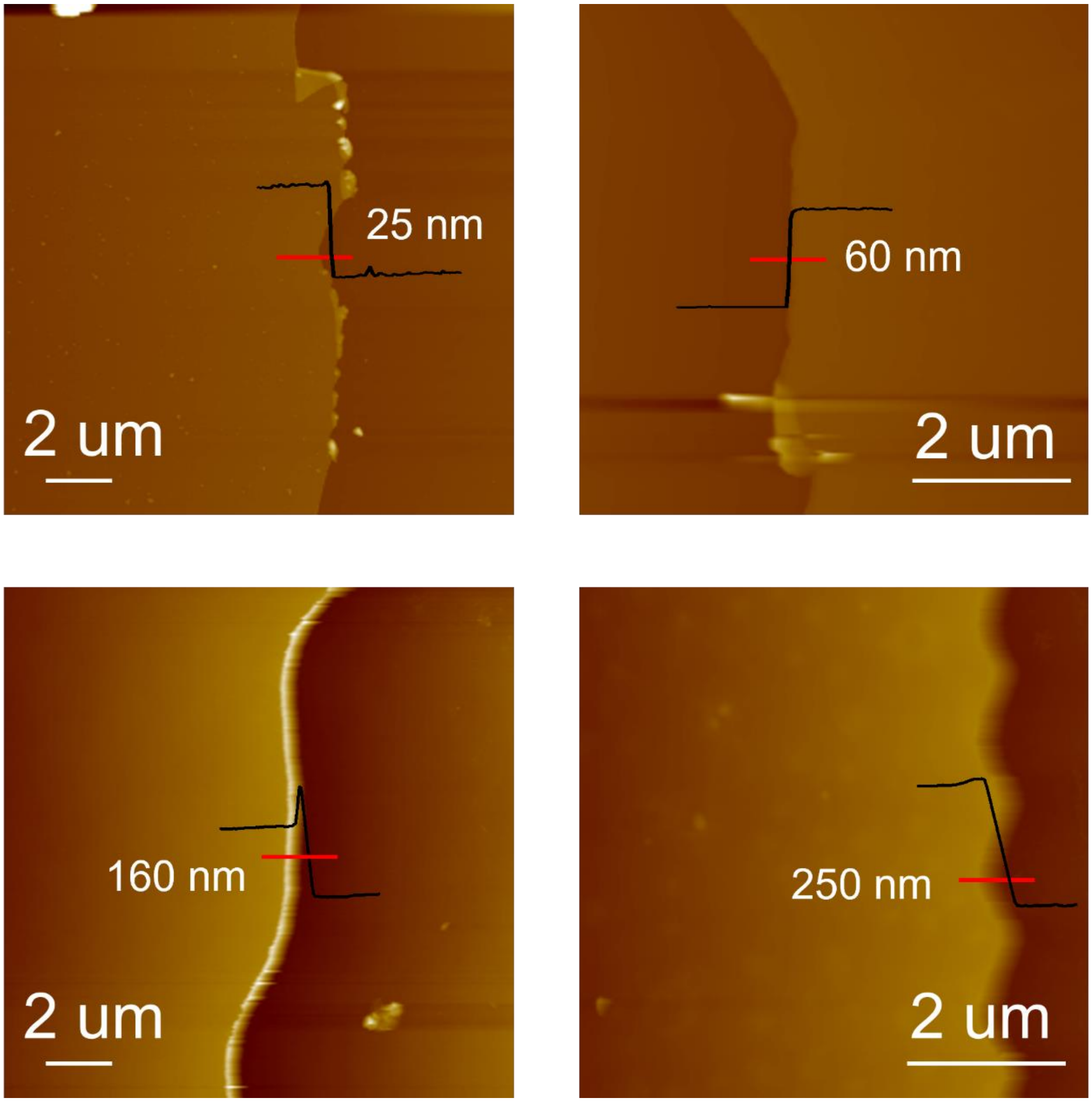

Supplementary Figure 3. AFM image of 2DP-F film ranging from 25nm to 250nm. Both films exhibited smooth and uniform surface.

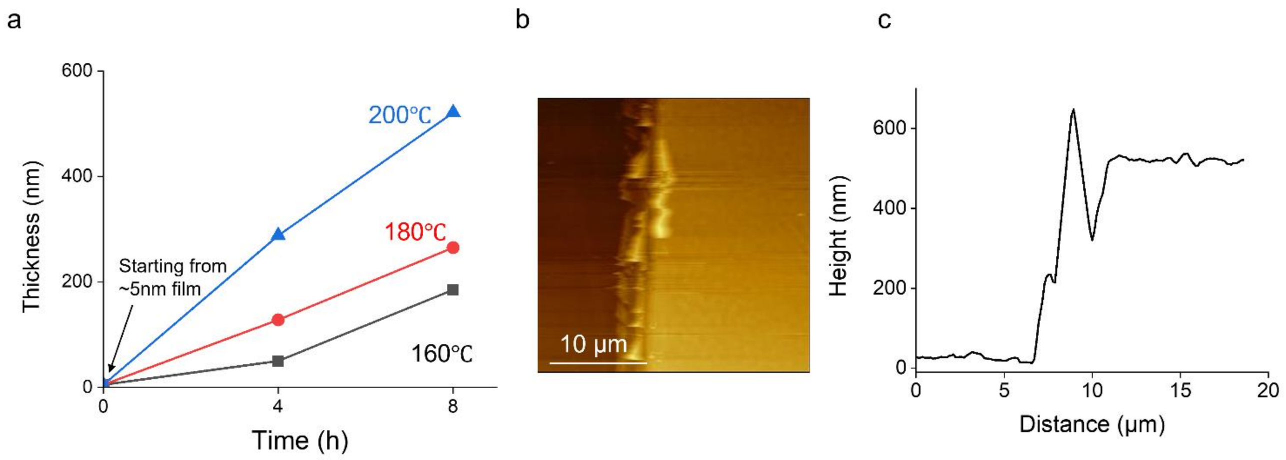

Supplementary Figure 4. (a) Thickness of the 2DP-F film as a function of growth time and temperature when using 5nm 2DP-F thin film as starting substrate and 3M AcOH (aq) as catalyst. (b) AFM image of a 530 nm thick 2DP-F film and (c) corresponding line profile.

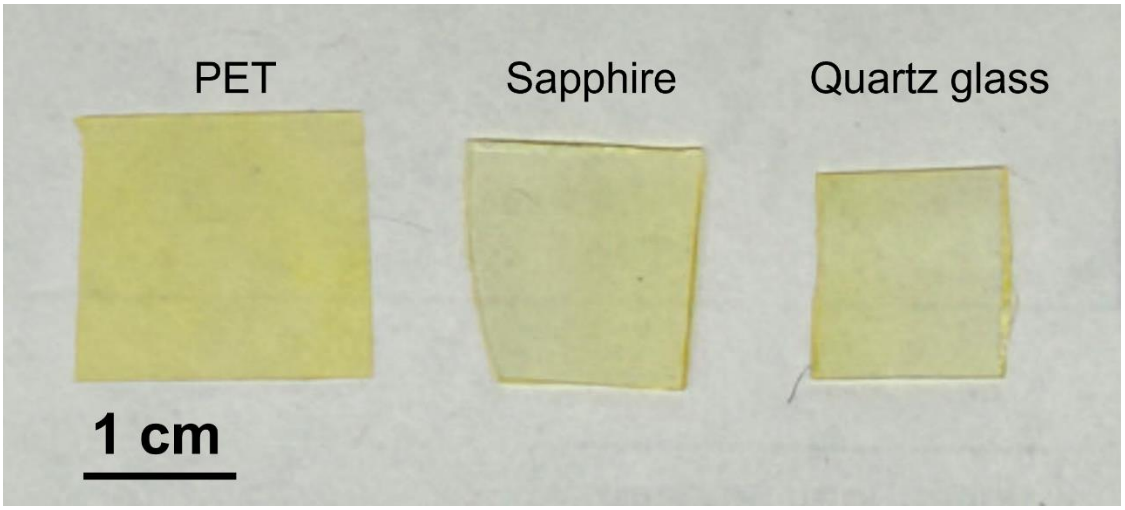

Supplementary Figure 5. Photograph of 2DP-F film grown in other transparent substrates.

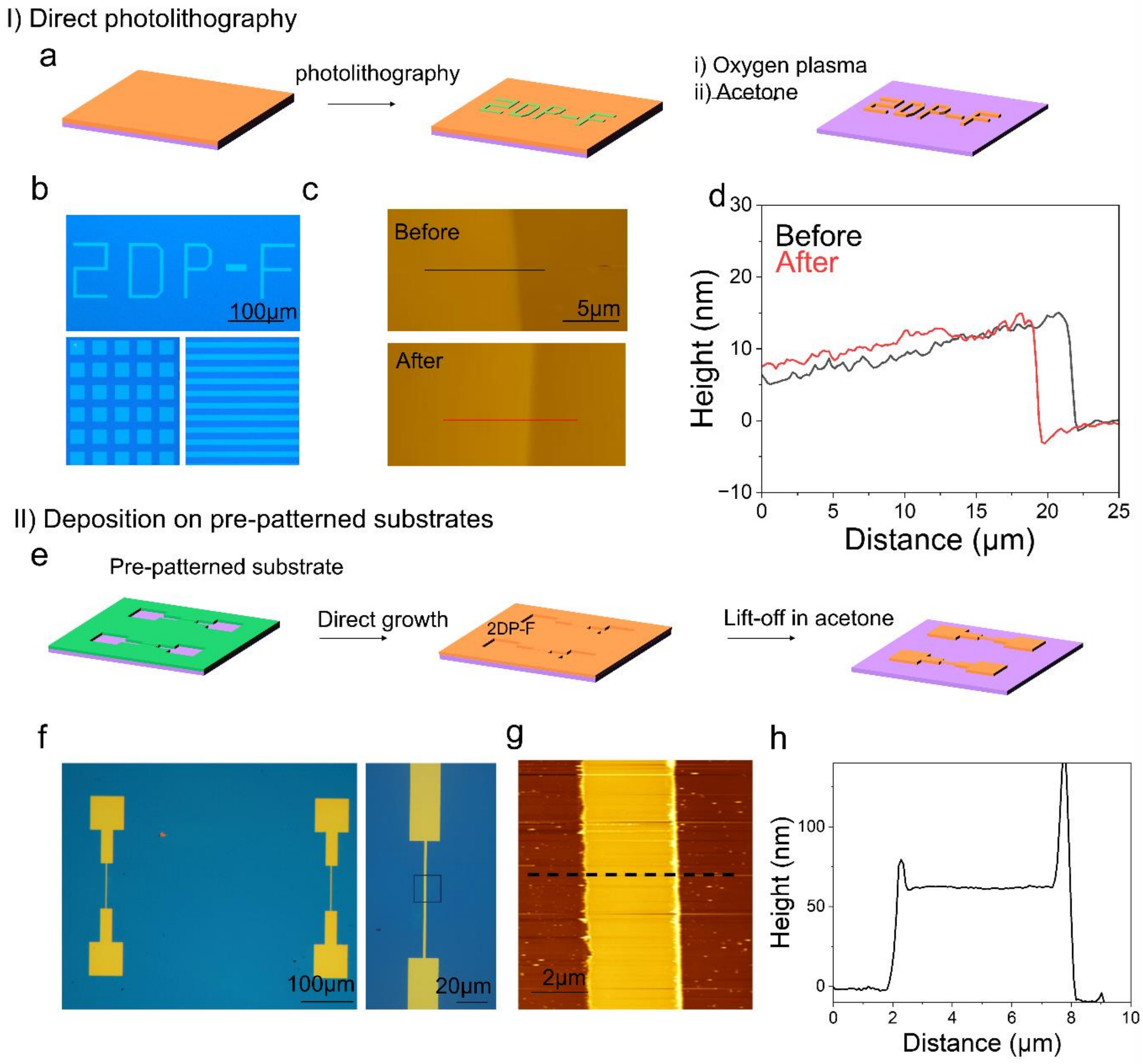

Supplementary Figure 6. (a) Schematical illustration of direct photolithography on 2DP-F films. (b) Optical image of patterned 2DP-F films. (c) AFM image of 2DP-F film before and after patterning and (d) Corresponding line profile. (e) Schematic illustration of patterning 2DP-F by direct deposition of 2DP-F on patterned substrates. (f) Optical image of the 2DP-F film after lift-off in acetone with ultrasonication. (g) AFM image of the area highlighted in black square and (h) Corresponding line profile.

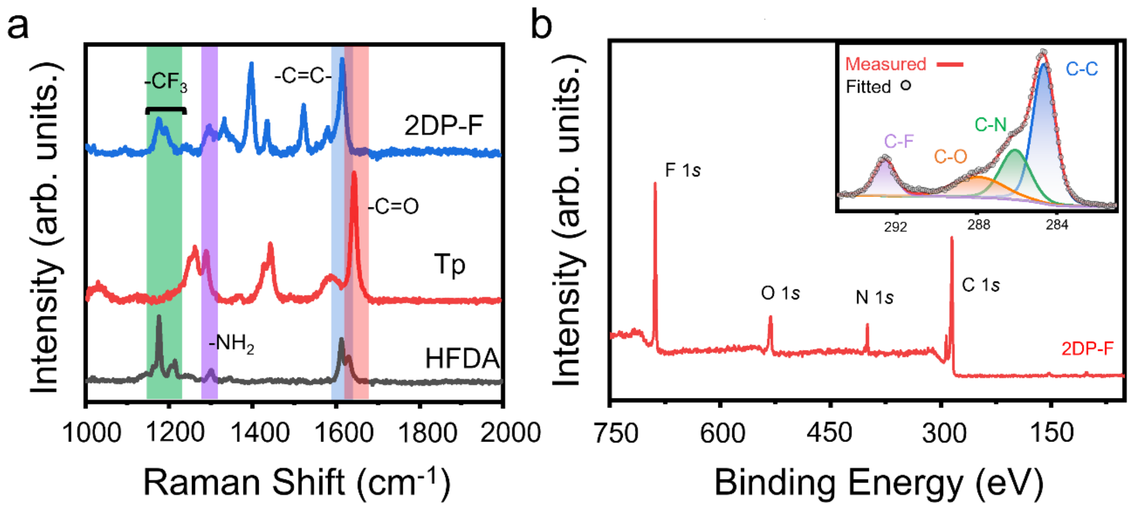

Supplementary Figure 7. (a) Raman spectra of 2DP-F film and monomers. (b) survey XPS of 2DP-F film. (inset: XPS spectrum of C1S signal from 2DP-F).

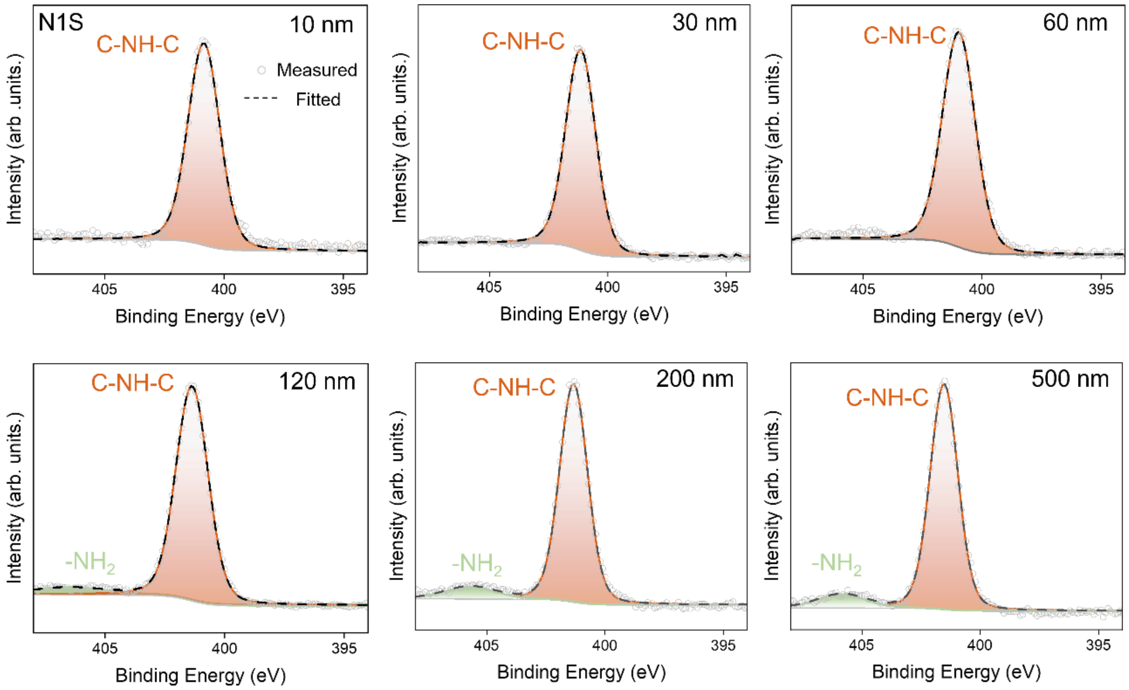

Supplementary Figure 8. Thickness-dependent XPS N1s spectra of 2DP-F films. The result indicates that some unreacted amino groups are present when the thickness is larger than 100 nm.

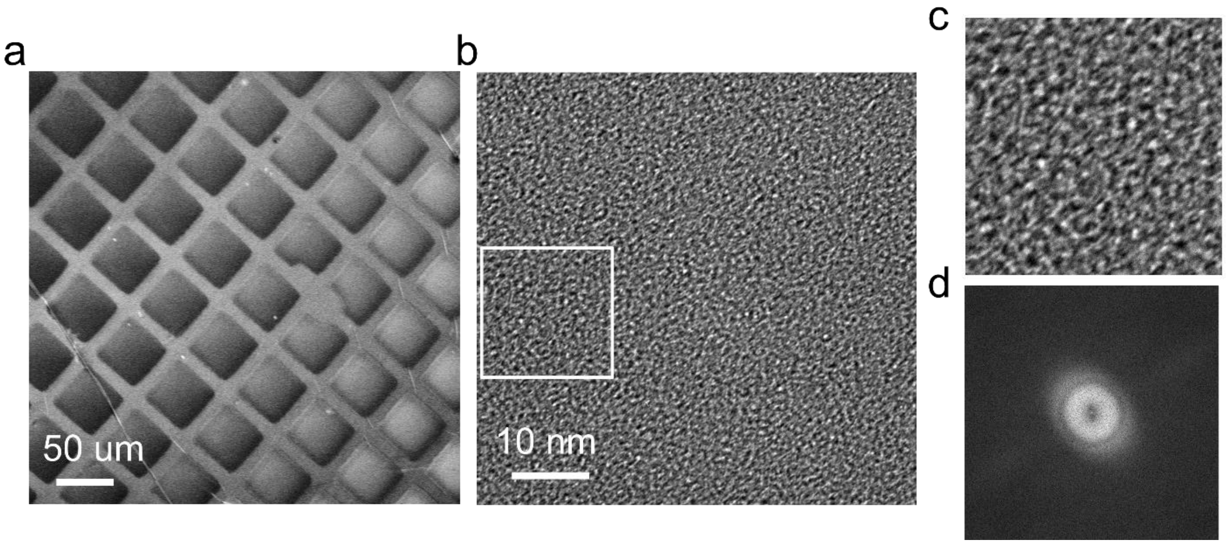

Supplementary Figure 9. (a). SEM image of a 20 nm 2DP-F suspended on a TEM grid. (b) High-resolution TEM image of suspended 2DP-F film. (c). A magnified view of the area indicated by the white box in b shows an amorphous structure. (d). FFT result of the area in (c) demonstrating a diffuse diffraction pattern of the amorphous structure.

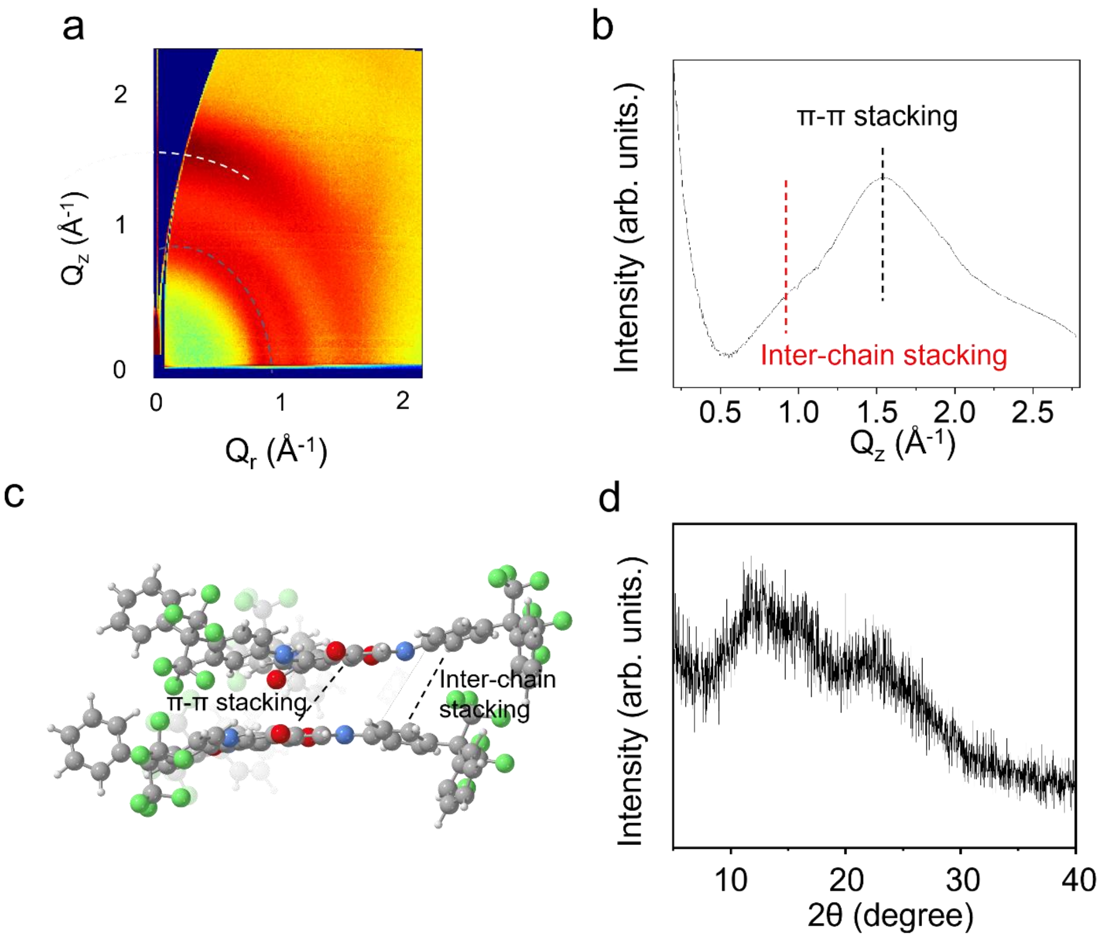

Supplementary Figure 10. (a)GIWAXS scattering 2D image and (b) its intensity profile near Q_r=0. (c) molecular structure of a building unit in 2DP-F. (d) PXRD of 2DP-F powder collected after the reaction.

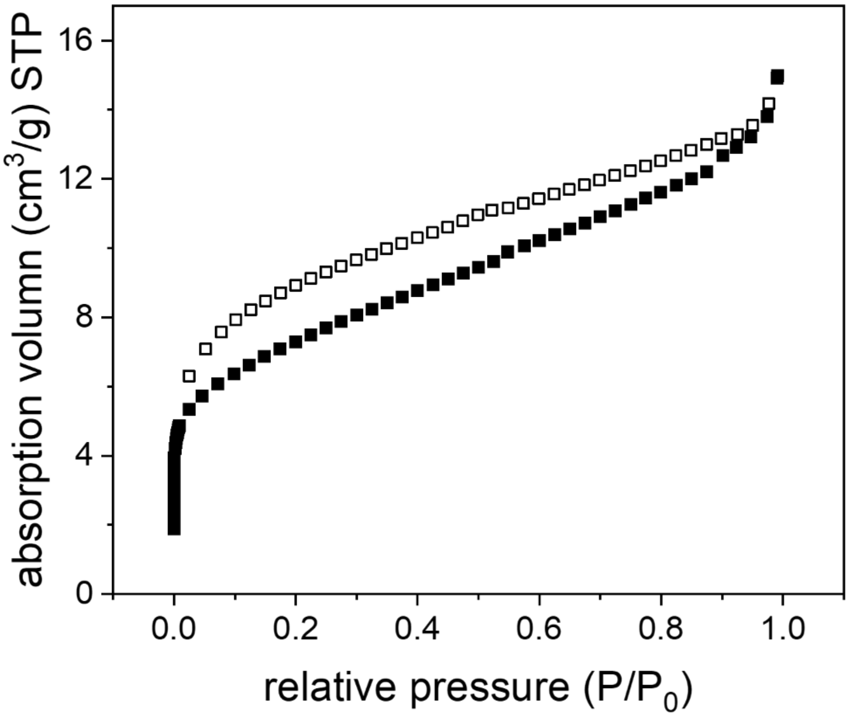

Supplementary Figure 11. Nitrogen sorption curves for 2DP-F powder.

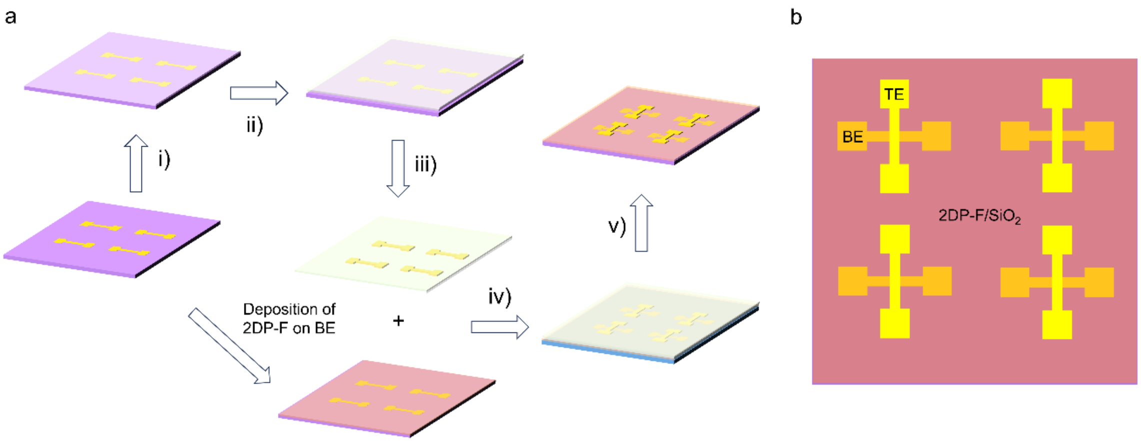

i) Spin coat PMMA; ii) thermal release tape; iii) Peel off electrode in the presence of 10% NaOH(aq);
iv) Put TRT/PMMA/Electrode on 2DP-F film; v) Remove TRT and PMMA by heating to 120℃ and acetone respectively.

Supplementary Figure 12. Schematical illustration of the “dry-transfer” method to fabricate MIM devices based on 2DP-F.

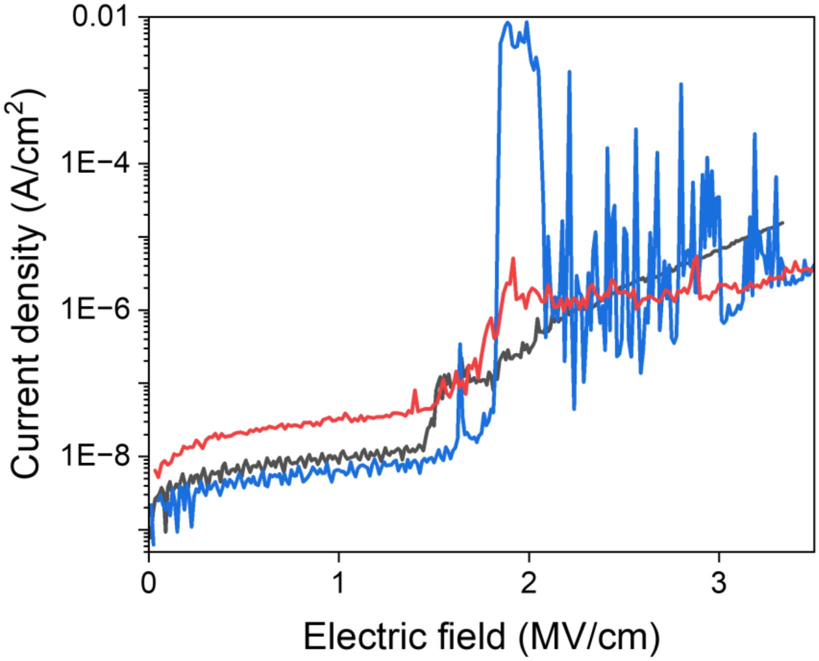

Supplementary Figure 13. Leakage current density versus the applied electrical field across the MIM devices based on 2DP-F with different thicknesses.

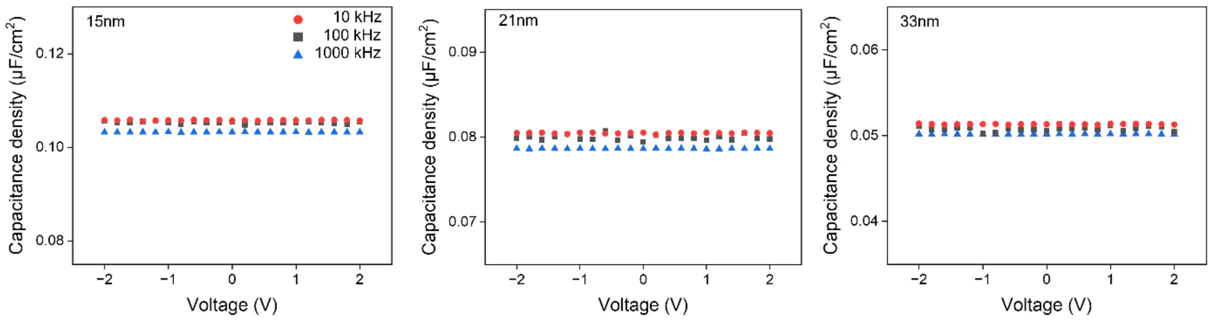

Supplementary Figure 14. Capacitance-voltage (C-V) characteristic of 2DP-F parallel capacitors with different thicknesses.

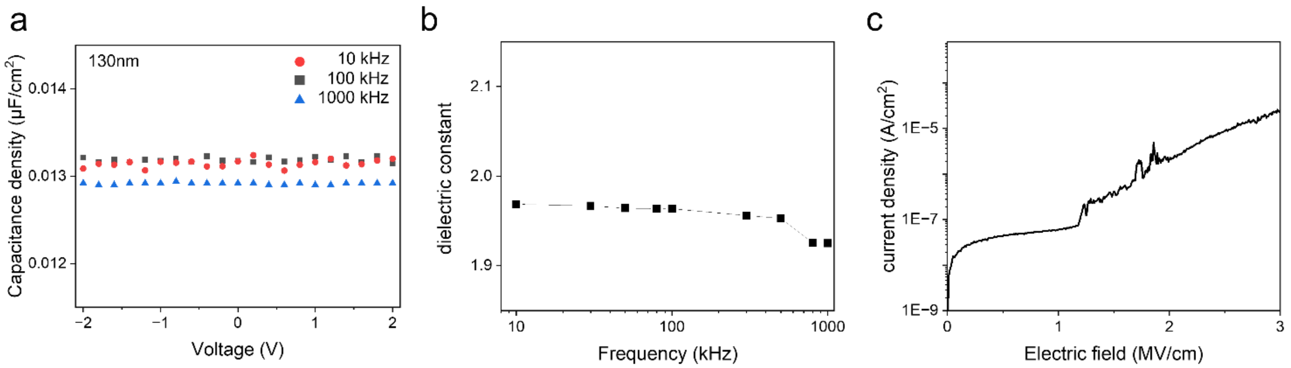

Supplementary Figure 15. (a) Capacitance-voltage (C-V) characteristic, (b) C-f characteristic, and (c) leakage current of 2DP-F MIM device prepared using the direct metal deposition method.

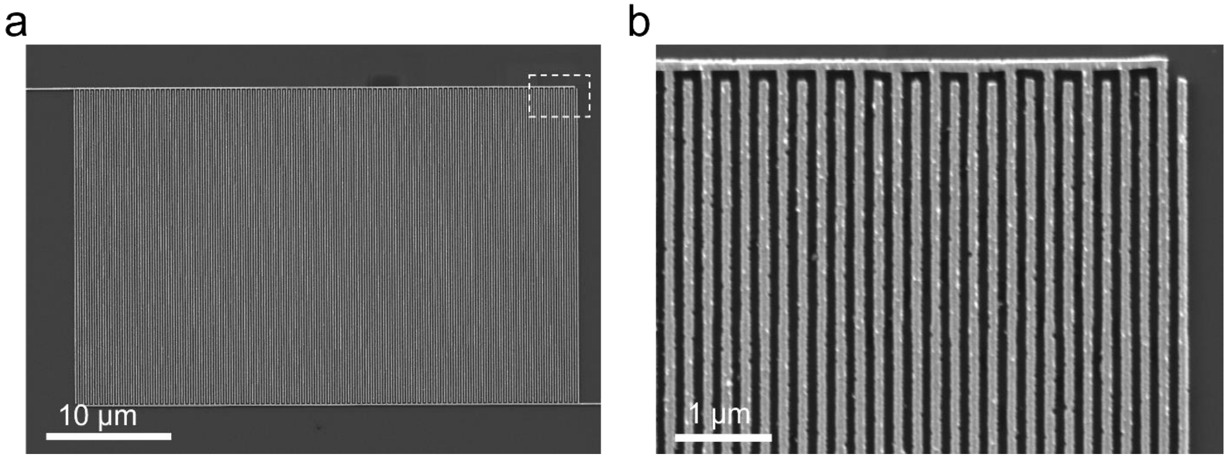

Supplementary Figure 16. (a). SEM image of the interdigital capacitors and (b) magnification of the area indicated by the white box in a.

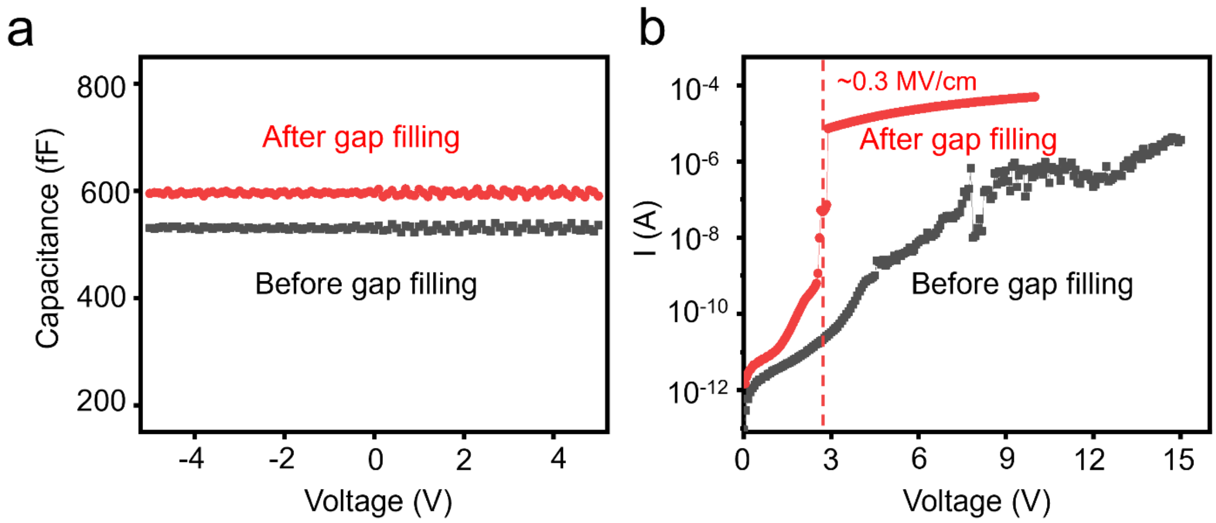

Supplementary Figure 17. (a). C-V characteristic of interdigitated capacitors before and after gap filling. (b) breakdown of the interdigitated capacitors.

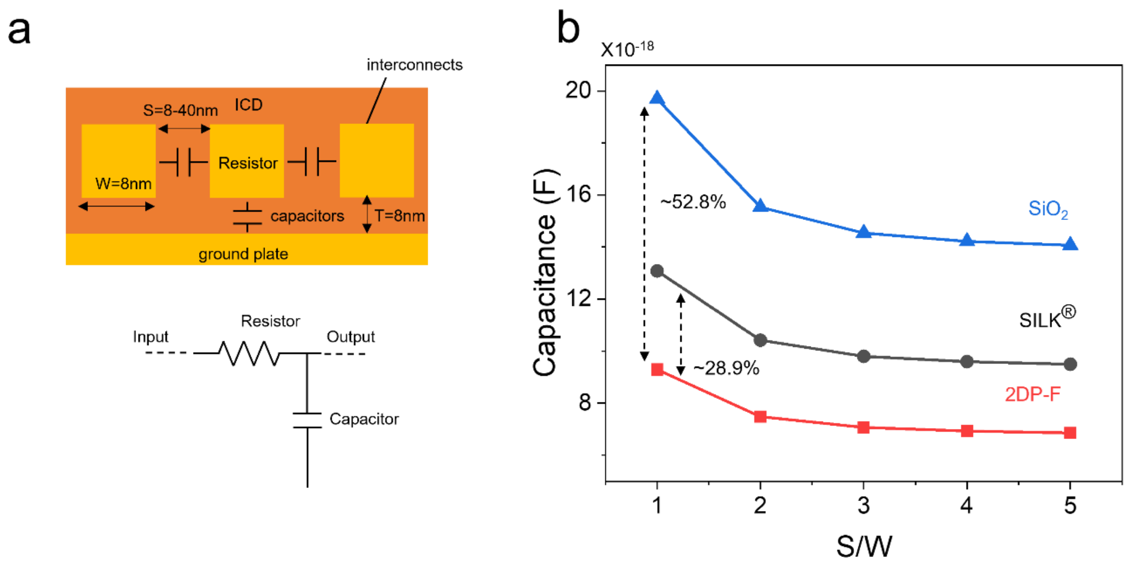

Supplementary Figure 18. (a). Schematic of a simplified Cu interconnect structure (top) and corresponding equivalent circuit used for the Silvaco TCAD simulation. (b) Interconnect parasitic capacitance reduction by substituting silicon oxide and SILK® with 2DP-F.

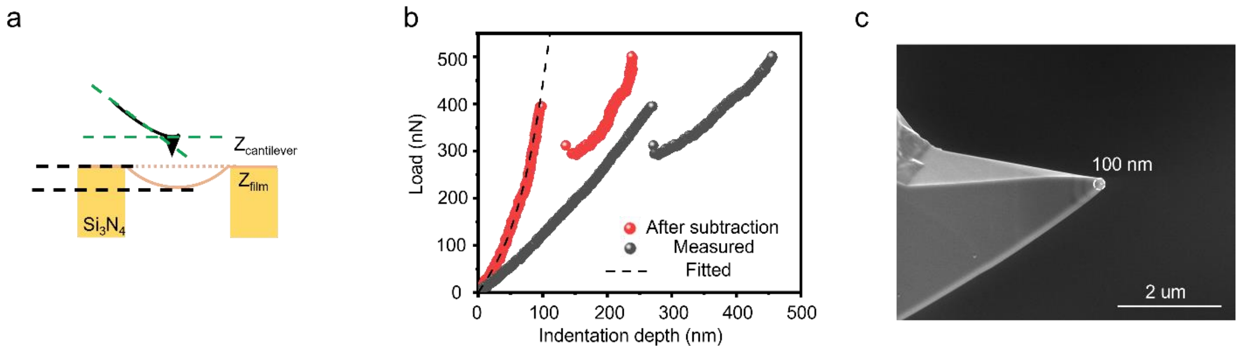

Supplementary Figure 19. **(**a). Schematic illustration of the indentation test. The displacement of the AFM tip includes two parts. (1) deformation of the AFM cantilever, and it can be expressed as $Z_{cantilever}$=F/Kc., where F is the load and Kc is the stiffness of the cantilever. (2) deflection of the thin film, and it can be expressed as Z_{film}=displacement- $Z_{cantilever}$, and the load-deflection curves of the film can be obtained by subtracting cantilever deformation, as illustrated in b. c. SEM image of the AFM tip.

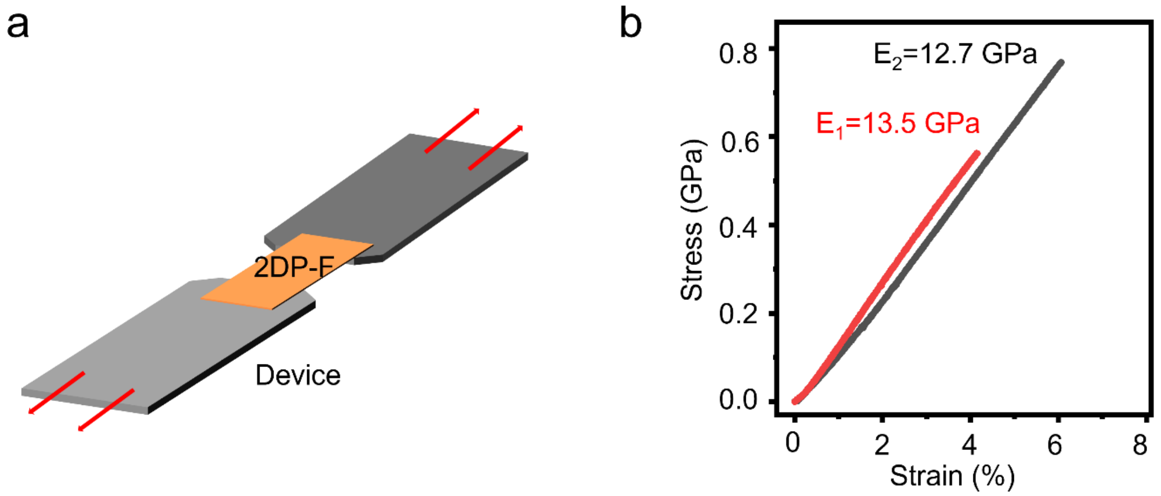

Supplementary Figure 20. (a) Schematic illustration of the tensile test of 2DP-F film. (b) Stran-stress curves of a 40 nm 2DP-F film. The highest Young's modulus reaches 13.5 GPa.

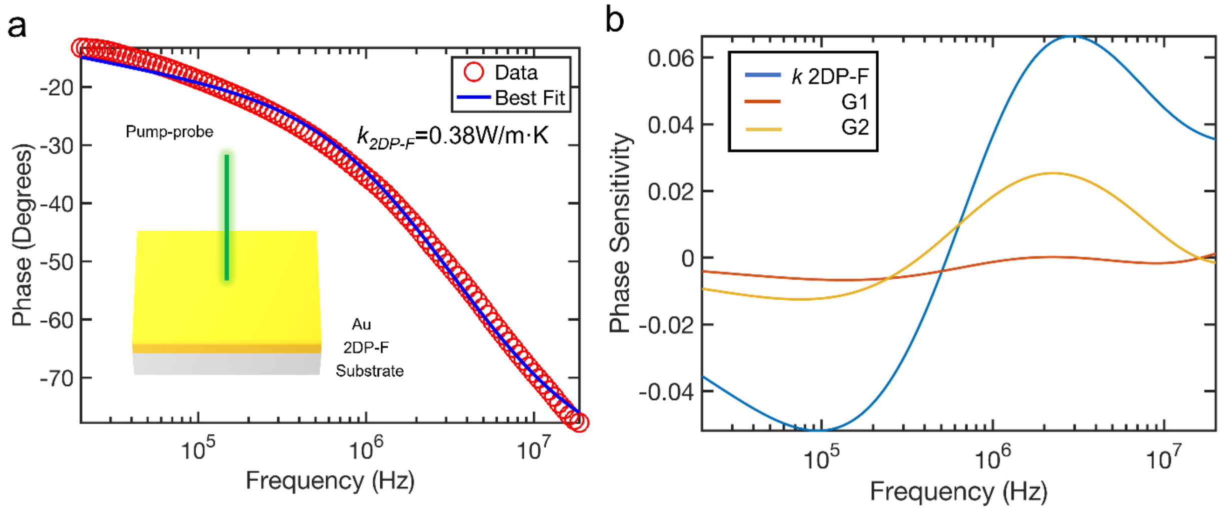

Supplementary Figure 21. (a) Phase lag vs. frequency data obtained from FDTR measurements shows a good approximation to the calculated best-fit curve. Each measurement is an average of three runs. (inset: schematic of FDTR measurement). (b) Sensitivity analysis of the thermal conductivity k of the COF, the thermal boundary conductance G1 between Au and the COF, and G2 between the COF and the substrate. k of the COF is highly sensitive throughout the frequency range of our measurement.

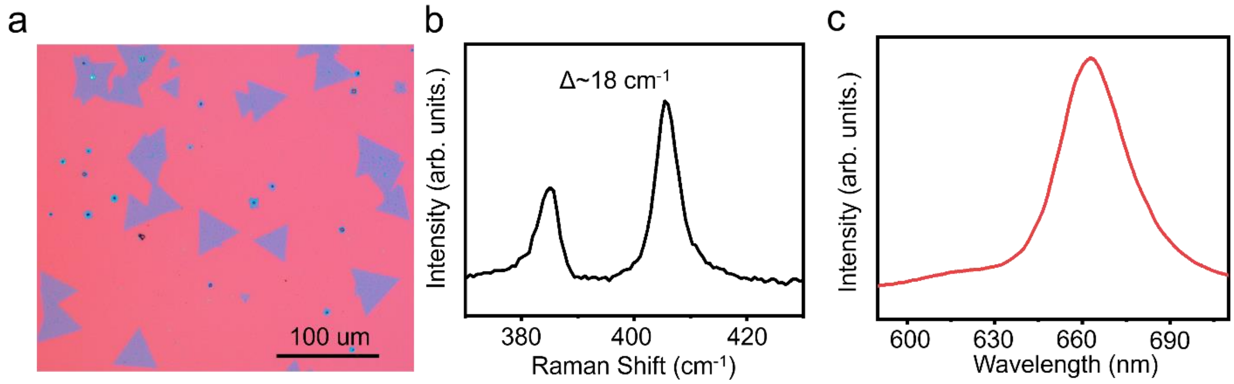

Supplementary Figure 22. (a) Optical image of CVD-grown MoS_2 crystals. (b) Raman and (c) PL spectrum of monolayer MoS_2.

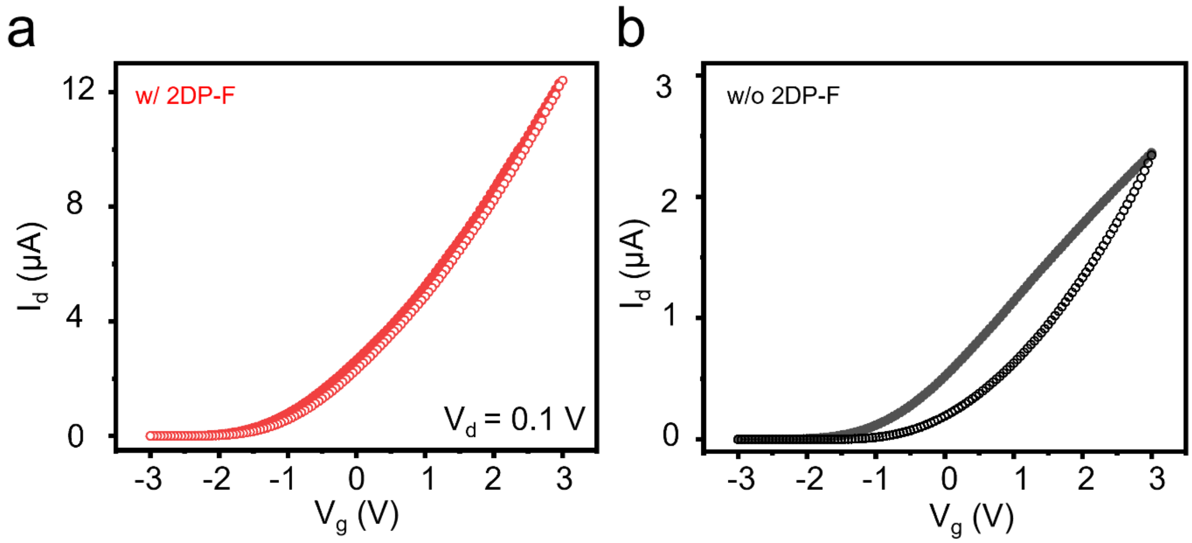

Supplementary Figure 23. Transfer curve of MoS_2 a. with and b. without 2DP-F film dielectric layer, demonstrating that 2DP-F significantly suppressed the hysteresis induced by oxides.

Category	Materials	k	YM(GPa)	Density (g/cm3)	Normalized YM	reference
MOFs	ZIF-67	2.39	3.79	0.94	4.03	[1]
	ZIF-8	2.23	3.15	0.96	3.28	
	ZIF-8	2.33	3	0.96	3.125	[2]
	HKUST-1	2.8	22	1.07	20.56	[3]
PSZs	PSZ-MFI film	2.7	53.9	1.76	30.625	[4]
	PSZ-FER crystal	1.78	49.4	/		
	PSZ MFI film on gold	1.71	54	1.76	30.625	[5]
Porous OSGs	22 C:H	2.25	4.5	0.7	6.428	[6]
	625-SiOC:H	2.2	4.5	0.9	5	
	186-SiOC:H	2.25	5.1	0.9	5.67	
	322-SiOC:H	2.2	5.4	0.9	6	
	94-SiOC:H	2	4.4	0.7	6.29	
	Si0.2C0.8:H#1	3.2	3.5	1.15	3.04	[7]
	Si0.2C0.8:H#2	3.2	4.8	1.15	4.12	
	Si0.2C0.8:H#3	2.85	6.8	1.15	5.91	
	SiOC:H#1	2.55	5.9	1.1	5.36	
	SiOC:H#2	2.6	5.7	1.1	5.18	
	SiOC:H#3	2.5	8.3	1.25	6.64	
	SiOC:H#4	2.5	8.7	1.25	6.96	
	c-T8B8	2.25	2.35	1.191	1.97	[8]
	c-T10B10	2.03	2.97	1.182	2.51	
	c-T12B12	1.83	3.35	1.176	2.85	
	c-T8PB8	2.30	2.5	1.141	2.19	
	c-T10PB10	2.10	2.4	1.125	2.13	
	c-T12PB12	1.93	2.43	1.119	2.17	
	c-T8F8	2.52	2.36	1.281	1.84	
	c-T10F10	2.33	2.51	1.274	1.97	
	c-T12F12	2.14	2.69	1.266	2.12	
	TmBPHF	2.1	2.02	1.21	1.67	[9]
	p-DBCOD-BCB	2.66	3.7			[10]

·

polymers	p-DBCOD-ene-BCB	2.54	3.8			
	PI-FH	2.05	2.11	1.42	1.49	11
	PI-FO	2.76	2.42	1.42	1.70	
	PI-FP	2.92	2.86	1.42	2.01	
	6FDA-PFODA-trans	2.42	2.5	1.246	2.00	12
	6FDA-PFODA-cis	2.44	3.1	1.249	2.48	
	PFODPA-TFMB	2.46	2.7	1.241	2.18	
	6FDA-co-PFODPA-PFODA	2.37	2.2	1.241	1.77	
	6FDA-TFMB	2.78	2.5	1.283	1.95	
	CYTOP®	2.1	1.3	2.03	0.64	13
2DPs	TAPB-TPOCx-COF	1.2	1.4	0.4	3.50	14.
	2DP-F	1.85	16.8	0.46	36.5	This work

Supplementary Table 1. Meta-analysis of other low-k dielectrics with reported Young's modulus

Supplementary References

1. Krishtab, M. *et al.* Vapor-deposited zeolitic imidazolate frameworks as gap-filling ultra-low-k dielectrics. *Nature Communications* **10**, 3729 (2019).
2. Eslava, S. *et al.* Metal-Organic Framework ZIF-8 Films As Low-κ Dielectrics in Microelectronics. *Chemistry of Materials* **25**, 27-33 (2013).
3. Babal, A.S., Chaudhari, A.K., Yeung, H.H.-M. & Tan, J.-C. Guest-Tunable Dielectric Sensing Using a Single Crystal of HKUST-1. *Advanced Materials Interfaces* **7**, 2000408 (2020).
4. Li, Z. *et al.* Mechanical and Dielectric Properties of Pure-Silica-Zeolite Low-k Materials. *Angewandte Chemie International Edition* **45**, 6329-6332 (2006).
5. Tiriolo, R. *et al.* Sub-Micrometer Zeolite Films on Gold-Coated Silicon Wafers with Single-Crystal-Like Dielectric Constant and Elastic Modulus. *Advanced Functional Materials* **27**, 1700864 (2017).
6. Zeng, M., Zhang, J. & Wang, Y. Investigation of the impact of annealing temperature on characteristics of porous SiOCH films. *Journal of Physics: Conference Series* **2563**, 012033 (2023).
7. Zhou, W. *et al.* Elastic properties of porous low-k dielectric nano-films. *Journal of Applied Physics* **110**, 043520 (2011).
8. Zhou, D.-L. *et al.* Linker engineering of larger POSS-based ultra-low-k dielectrics toward outstanding comprehensive properties. *Giant* **14**, 100146 (2023).
9. Qian, C. *et al.* Facile Strategy for Intrinsic Low-k Dielectric Polymers: Molecular Design Based on Secondary Relaxation Behavior. *Macromolecules* **52**, 4601-4609 (2019).
10. Feng, Y., Jin, K., Guo, J. & Wang, C. All-carbocycle hydrocarbon thermosets with high thermal stability and robust mechanical strength for low-k interlayer dielectrics. *Polymer Chemistry* **12**, 4812-4821 (2021).
11. Sun, Y. *et al.* Preparation and Characterization of Intrinsic Low-κ Polyimide Films. *Polymers* **13**, 4174 (2021).
12. Peng, W., Lei, H., Qiu, L., Bao, F. & Huang, M. Perfluorocyclobutyl-containing transparent polyimides with low dielectric constant and low dielectric loss. *Polymer Chemistry* **13**, 3949-3955 (2022).
13. Fong, N.R., Berini, P. & Tait, R.N. Mechanical Properties of Thin Free-Standing CYTOP Membranes. *Journal of Microelectromechanical Systems* **19**, 700-705 (2010).
14. Shao, P. *et al.* Flexible Films of Covalent Organic Frameworks with Ultralow Dielectric Constants under High Humidity. *Angewandte Chemie International Edition* **57**, 16501-16505 (2018).
